# 1,8-Cineole (Eucalyptol) in Neurodegeneration: From Pharmacological Mechanisms to Therapeutic Applications

**DOI:** 10.3390/cells15191748

**Published:** 2026-09-24

**Authors:** Tamara Janković, Kristina Pilipović, Nika Gržeta Krpan, Jelena Rajič Bumber, Silvestar Mežnarić, Ana Pelčić, Petra Dolenec, Tea Ćelić, Jasenka Mršić-Pelčić

**Affiliations:** 1 Department of Basic and Clinical Pharmacology and Toxicology, Faculty of Medicine, University of Rijeka, 51000 Rijeka, Croatia; kristina.pilipovic@medri.uniri.hr (K.P.); nika.grzeta.krpan@medri.uniri.hr (N.G.K.); jelena.rajic@medri.uniri.hr (J.R.B.); silvestar.meznaric@medri.uniri.hr (S.M.); petra.dolenec@medri.uniri.hr (P.D.); tcelic@student.uniri.hr (T.Ć.); jasenka.mrsic.pelcic@medri.uniri.hr (J.M.-P.); 2 Department of Emergency Medicine, Clinical Hospital Centre Rijeka, 51000 Rijeka, Croatia; ana.pelcic@gmail.com

**Keywords:** terpenoids, 1,8-cineole, neurodegeneration, neuroinflammation, oxidative stress, blood–brain barrier, phytomedicine

## Abstract

**Highlights:**

**What are the main findings?**

- 1,8-cineole provides multitarget neuroprotection by modulating inflammation, oxidative stress, apoptosis, neurotransmission, and pain signaling.
- It demonstrates neuroprotective potential in preclinical models of Alzheimer’s disease, Parkinson’s disease, stroke, anxiety, and depression.

**What are the implications of the main findings?**

- Its multitarget action makes 1,8-cineole a promising candidate for future neuroprotective treatments.
- Pharmacokinetic limitations highlight the need for advanced drug-delivery systems, while limited clinical evidence indicates a gap in translation to human studies.

**Abstract:**

Neurodegenerative diseases represent an enormous global burden, and due to the lack of effective therapies, the number of affected individuals continues to rise, placing a significant pressure on patients and their families. Phytochemicals such as terpenes are a group of compounds that have the potential to provide long-term neuroprotective effects by targeting multiple pathobiological pathways and supporting the brain’s natural defense mechanisms. 1,8-cineole (eucalyptol) is a monoterpene known for its anti-inflammatory and antioxidant properties, mainly utilized in the respiratory system. However, due to its ability to cross the blood–brain barrier, beneficial effects have been observed in the central nervous system. Here we examine the pharmacological mechanisms and neuroprotective potential of 1,8-cineole in the nervous tissue. Studies have shown that 1,8-cineole has the potential to suppress neuroinflammation and reduce glial activation, activate antioxidant defense mechanisms and scavenge reactive oxygen species. Additionally, 1,8-cineole has been shown to modulate central neurotransmission and synaptic plasticity, inhibit apoptotic neuronal death and regulate ion channels involved in pain modulation. This review aims to examine the therapeutic potential of 1,8-cineole in the most prevalent neurodegenerative diseases in the population, such as Alzheimer’s and Parkinson’s diseases, as well as brain injuries, including ischemic stroke and traumatic brain injury, which may lead to neurodegeneration. We also explore the potential benefits of 1,8-cineole for anxiety and depression, which occur as early symptoms or common complications of neurodegenerative diseases. Given the need for novel therapeutic approaches, combination therapies that utilize the therapeutic potential of phytochemicals, such as 1,8-cineole, may enable more effective targeting of the multiple, interconnected pathobiological processes underlying neurodegenerative diseases.

## 1. Introduction

Neurodegenerative diseases, such as Alzheimer’s disease (AD), Parkinson’s disease (PD), amyotrophic lateral sclerosis (ALS), Huntington’s disease (HD), frontotemporal lobar dementia (FTLD) and Lewy body disease [1] represent an enormous global health burden. It is estimated that more than 57 million people are affected by these conditions, with this number projected to double every 20 years [2]. Numerous common pathological mechanisms have been identified in the development of neurodegenerative diseases, such as: biological ageing, genetic predisposition, aberrant protein folding and accumulation, mitochondrial impairment, chronic neuroinflammation, impaired axonal transport, microbiome dysbiosis, synaptic and circuitry dysfunction and targeted apoptosis and necrosis [1]. Currently, therapeutic options are limited and focused solely on an individual pathobiological pathway, making them insufficient to halt disease progression and reduce the increase in numbers of affected individuals.

Due to their complex mechanisms of action, phytochemicals such as polyphenols and terpenes act on multiple molecular targets and represent potential candidates for novel and combined therapies [3]. Moreover, continuous exposure to phytochemicals throughout life, combined with healthy lifestyle habits [4], has been recognized as neuroprotective [5]. A long-term diet rich in phytochemicals may provide sustained antioxidant protection, help prevent the development of chronic inflammation and support the brain’s endogenous defense mechanisms, potentially for decades before the first symptoms of neurodegenerative diseases appear [6].

Phytochemicals, including terpenoids, are increasingly recognized for their potential to modulate oxidative stress through both direct and indirect antioxidant mechanisms [7]. These mechanisms include the scavenging of reactive oxygen species (ROS), inhibition of lipid peroxidation, and modulation of endogenous antioxidant defense pathways. Among these, the Nrf2/Keap1 pathway represents a major cellular mechanism involved in maintaining redox homeostasis by regulating the expression of antioxidant and cytoprotective genes. Such mechanisms may be particularly relevant in the nervous system, where high metabolic activity and oxygen consumption make neuronal tissue highly susceptible to oxidative damage [8].

1,8-cineole (eucalyptol; 1,3,3-trimethyl-2-oxabicyclo [2.2.2]octane) is a natural monocyclic monoterpene compound present as the most abundant compound in the eucalyptus plant (*Eucalyptus spp.*) [9], where it serves as a natural defense toxin against a herbivore called the brushtail possum (*Trichosurus vulpecula*) [10]. 1,8-cineole itself is an achiral, meso molecule due to the internal symmetry of its bicyclic skeleton [11,12] (Figure 1), whereas several of its oxidative (phase I) metabolites are chiral, occurring as mixtures of both enantiomers in variable, non-racemic ratios that reflect the stereoselectivity of the underlying enzymatic biotransformation [13,14,15].

Furthermore, it is also a main component in numerous Mediterranean plants and their essential oils, such as bay laurel (*Laurus nobilis* L.) [16,17], rosemary (*Rosmarinus officinalis* L.), thyme (*Thymus vulgaris* L.) [18] and sage (*Salvia officinalis* L.) [19] with concentrations varying, depending on the extraction method and plant origin. The therapeutic potential of 1,8-cineole has long been recognized. Australian Aboriginal Dharawal people have traditionally been using essential oils containing 1,8-cineole as a main compound for its anti-inflammatory properties in numerous conditions, like bladder or eye inflammation and fever [20]. Nowadays, in both Western and Eastern medicine, it is used as a key component of pharmaceutical formulations for the treatment of acute and chronic bronchitis and sinusitis [21]. Additionally, it is widely used as an excipient in the pharmaceutical and cosmetic industries, due to its favorable safety profile and pleasant taste and aroma [22]. In the pharmaceutical industry, 1,8-cineole is incorporated into a variety of pharmaceutical formulations. For oral administration, it is available in soft gastro-resistant capsules, which are used for the treatment of bronchitis, sinusitis, and chronic obstructive pulmonary disease [23,24]. For topical application, 1,8-cineole is formulated as an ointment intended for application to the chest and neck, from where it is absorbed through the skin [25]. It is also incorporated into mouthwashes for the control of dental plaque and gingivitis [26,27]. According to the Scientific Committee on Food of the European Commission and the U.S. Food and Drug Administration [28,29], 1,8-cineole is considered safe for use in various over-the-counter preparations, including throat and cough lozenges. In addition, it is widely used in toothpastes as a flavoring, adjuvant [30] and antiseptic agent [31]. In the cosmetic industry, 1,8-cineole is used primarily as a component of fragrance compositions [32]. It is generally recognized as safe as a food additive by the Expert Panel of the Flavor and Extract Manufacturers Association and is approved as a flavoring substance in the European Union. Combined with its pharmaceutical applications, this explains its frequent use in nutraceutical and parapharmaceutical formulations [33,34].

Due to its low molecular weight and lipophilic nature this monoterpene readily crosses the blood–brain barrier (BBB) [35,36,37], thereby making it particularly well suited for central nervous system (CNS) targeting. Given these favorable pharmacokinetic properties, extensive research has investigated its neuroprotective, anti-inflammatory, and antioxidant effects across various neurodegenerative and neurological disorders [38]. Indeed, a growing body of research confirms that it has therapeutic neuroprotective potential in CNS disorders, including neuropathic pain [39], AD [40] and ischemic injury following stroke [41,42]. Multiple studies have demonstrated that 1,8-cineole exerts these neuroprotective effects through diverse cellular and molecular pathways. Evidence suggest that it may suppress neuroinflammation [43,44,45] and excessive glial activation [44,45,46,47], enhance antioxidant defense mechanisms [35,41,47,48,49] and scavenge ROS, thereby protecting neurons from oxidative and inflammatory damage. Furthermore, it may modulate central neurotransmission and synaptic plasticity [35,50,51,52,53,54,55,56,57], inhibit apoptotic neuronal death [58,59], and regulate ion channels involved in pain signaling [39,60].

The literature considered in this narrative review was identified through searches of PubMed, Web of Science, and Google Scholar, without restriction on the publication year. The search was guided by combinations of the following keywords and Boolean operators: (″1,8-cineole″ OR ″eucalyptol″) AND (″pharmacokinetics″ OR ″absorption″ OR ″distribution″ OR ″metabolism″ OR ″bioavailability″ OR ″blood-brain barrier″ OR ″neuroprotection″ OR ″neurodegeneration″ OR ″oxidative stress″ OR ″antioxidant activity″ OR ″neuroinflammation″ OR ″glial activation″ OR ″neurotransmission″ OR ″synaptic plasticity″ OR ″apoptosis″ OR ″neuronal death″ OR ″ion channels″ OR ″pain″ OR ″Alzheimer’s disease″ OR ″Parkinson’s disease″ OR ″ischemic stroke″ OR ″traumatic brain injury″ OR ″anxiety″ OR ″depression″). Additional relevant publications were identified by examining the reference lists of selected articles and relevant review papers. The literature was considered based on its relevance to the scope and objectives of the present narrative review, with particular emphasis on the pharmacokinetic characteristics, pharmacological mechanisms, neuroprotective effects, and therapeutic potential of 1,8-cineole.

The aim of this narrative review is to comprehensively synthesize current evidence on the pharmacological and neuroprotective mechanisms of 1,8-cineole and critically evaluate its therapeutic potential in neurological disorders. Particular emphasis is placed on preclinical evidence in the most prevalent neurodegenerative diseases in the population, such as AD and PD, as well as acute neurological conditions such as ischemic stroke and traumatic brain injury (TBI), which may contribute to the development of neurodegeneration. Furthermore, the review addresses the potential benefits of 1,8-cineole in anxiety and depression, which may occur as early manifestations or common comorbidities of neurodegenerative disorders.

## 2. Pharmacokinetics and Blood–Brain Barrier Permeability of 1,8-Cineole

The pharmacological relevance of 1,8-cineole is largely attributed to its favorable physicochemical properties, including its relatively low molecular weight (154.25 g/mol) and moderate lipophilicity, expressed as XlogP (the log_10_ value of the calculated octanol/water partition coefficient) of approximately 2.5–2.8 [61]. These characteristics facilitate rapid membrane permeation and efficient absorption through multiple routes of administration, including oral, inhalational, and transdermal delivery, thereby contributing to its systemic distribution [36,62]. A particularly important pharmacological feature of 1,8-cineole is its ability to cross the BBB and penetrate into the CNS. This property has generated considerable interest in the potential of its therapeutic applications for the treatment of neurological and neurodegenerative disorders [37,63].

### 2.1. Absorption and Distribution

Following oral administration, 1,8-cineole is rapidly absorbed from the gastrointestinal tract, consistent with its lipophilic nature and high membrane permeability [64]. Human studies indicate that peak plasma concentrations are generally achieved within 45 min—2.5 h, although the rate and extent of absorption may vary with formulation, food intake, and individual metabolic characteristics. These values are consistent with the 0.5–1 h range reported in animal studies. Reported terminal half-life values span from under an hour to several hours across species and administration routes, heavily influenced by sex-specific adipose distribution, dose formulation, and redistribution dynamics [36,62,64,65]. Oral bioavailability is considered relatively high despite first-pass hepatic metabolism. However, the main challenges for 1,8-cineole bioavailability are its high volatility, poor water solubility, and limited formulation stability, which can reduce dosing precision and complicate oral or inhaled delivery. Various formulation strategies, including lipid-based systems and nanoencapsulation, have been shown to optimize stability and bioavailability, and may further improve systemic exposure by enhancing solubility and potentially reducing presystemic metabolism [66,67,68].

Inhalation is another clinically relevant route of administration that enables rapid systemic absorption. Following inhalational administration, peak plasma concentrations are attained at approximately 18 min, with an elimination half-life of approximately 100–110 min [62]. The extensive surface area and rich vascularization of the lungs facilitate efficient entry into the systemic circulation while partially bypassing first-pass hepatic metabolism. This route enables a faster onset of action compared with oral administration and has traditionally been used in the treatment of respiratory disorders. Importantly, inhalation also enables rapid delivery of 1,8-cineole to the CNS [69,70,71]. In contrast, transdermal and mucosal delivery lead to pronounced local accumulation with minimal systemic exposure. Ex vivo skin diffusion studies have shown that 1,8-cineole acts as a penetration enhancer by disordering *stratum corneum* lipids and increasing drug partitioning into the skin, thereby improving percutaneous permeability [72].

Once absorbed, 1,8-cineole is distributed into highly perfused organs, including the lungs, liver, kidneys and brain, while its affinity for lipid phases may also permit transient partitioning into lipid-rich tissues. Another distinctive feature of 1,8-cineole pharmacokinetics is its pulmonary elimination. Approximately 20% of the oral dose reaches the peripheral airways and is eliminated via exhalation. This provides a distinct clinical advantage by delivering the volatile organic compound directly to the paranasal sinuses and upper respiratory tract from the luminal side [64,70,71].

### 2.2. Hepatic Metabolism and Elimination Pathways of 1,8-Cineole

The metabolism of 1,8-cineole occurs predominantly in the liver and involves the cytochrome P450 enzyme system. CYP3A4/5 and CYP2B6 have been identified as major contributors, although other CYP isoforms may also contribute to its oxidative biotransformation. Metabolic investigations have identified approximately ten metabolites, with α2-hydroxy-1,8-cineole described as predominant. Subsequent phase II conjugation, particularly glucuronidation of hydroxylated metabolites, increases their water solubility and facilitates renal elimination. The metabolic profile may also exhibit time- and concentration-dependent characteristics, suggesting that enzymatic capacity and substrate concentration can influence the disposition of 1,8-cineole and its metabolites [15,62,63].

1,8-cineole elimination occurs through multiple pathways. Renal excretion of polar and conjugated metabolites represents the most clinically important clearance mechanism, whereas a proportion of the absorbed compound is eliminated unchanged through exhalation because of its volatility [15,64]. Consequently, hepatic metabolic conversion serves as the primary rate-limiting step in systemic elimination, producing a broad range in terminal clearance rates across different experimental models [36,62,64,65].

### 2.3. Blood–Brain Barrier Permeability of 1,8-Cineole

An important pharmacokinetic characteristic of 1,8-cineole is its capacity to reach the CNS. Its small molecular size, moderate lipophilicity, and lack of a permanent charge at physiological pH are consistent with efficient BBB permeation. Experimental studies have detected 1,8-cineole in brain tissue following systemic administration, while reported central pharmacological effects, including anti-inflammatory, anxiolytic, anticonvulsant, analgesic, and cognitive effects, provide additional functional evidence that biologically relevant concentrations reach the CNS [38]. Beyond its intrinsic ability to cross the BBB, emerging evidence suggests that it may also transiently modulate BBB permeability. Experimental observations of increased Evans Blue extravasation and ultrastructural alterations in endothelial cells and tight junction architecture have been interpreted as evidence of a temporary “opening effect” on the BBB [38]. Such effects are of particular interest from a drug-delivery perspective because transient modulation of barrier integrity could potentially improve CNS penetration by therapeutic compounds that otherwise exhibit limited brain access [38,73]. However, this phenomenon must be interpreted with caution as the disruption of barrier integrity exists on a continuum with neurotoxicity. Namely, while low, controlled exposures may yield benign, reversible permeabilization, higher concentrations risk unselective influx of plasma proteins and circulating neurotoxins, leading to localized edema or neuroinflammation. Current evidence regarding the safety window and reversibility kinetics remains limited and it is not clear whether this effect represents a controlled therapeutic mechanism or an early sign of concentration-dependent endothelial cytotoxicity.

## 3. Cellular and Molecular Mechanisms of 1,8-Cineole Action in the Central Nervous System

1,8-cineole exerts neuroprotective effects through several interconnected mechanisms. Numerous pathways underlying its antioxidant and anti-inflammatory effects have been described in various tissues [63]. However, the following section focuses exclusively on its effects within neural tissue of the central and peripheral nervous systems (Figure 2).

### 3.1. Suppression of Neuroinflammation and Glial Activation

Neuroinflammation is characterized by a complex cascade of cellular reactions in the brain and spinal cord that are regulated primarily by microglia, astrocytes, endothelial cells, and infiltrating immune cells [74]. The physiological role of neuroinflammation is to promote the repair of damaged brain tissue. However, chronic neuroinflammation can establish a vicious cycle that promotes neurodegeneration and neuronal cell death, both of which are strongly associated with the development and progression of neurodegenerative diseases [75].

The immune response in the brain often begins with the activation of microglia. Microglia are resident innate immune cells that, under physiological conditions, continuously monitor the brain parenchyma to maintain homeostasis. Damage-associated molecular patterns, such as the intracellular energy carrier ATP, released into the in the extracellular environment act as chemical signals that, by binding to receptors on microglia, initiate a signaling cascade that transforms them from their resting state into an activated, phagocytic phenotype [76]. These activated microglia are transiently beneficial for brain tissue repair by facilitating the clearance of cellular and molecular debris. However, prolonged microglial activation contributes to detrimental chronic neuroinflammation through the production of cytotoxic mediators, including interleukin-1β (IL-1β), nitric oxide, ROS, and tumor necrosis factor-α (TNF-α) [77,78]. Chronically activated pro-inflammatory microglia have been associated with the progression of neurodegenerative disorders, such as chronic traumatic encephalopathy [79], ALS [80,81], AD and PD [80,82]. More specifically, in AD, microglia were found adjacent to amyloid-beta (Aβ) plaques and neurofibrillary tangles; in ALS, in the vicinity of the degenerated motor neurons and in PD, reactive microglia were detected *post mortem* in the *substantia nigra* [75,83,84,85].

In contrast, microglia can also adopt an anti-inflammatory phenotype characterized by the release of anti-inflammatory cytokines, such as interleukin-10 (IL-10), transforming growth factor-β, and interleukin-6 (IL-6), as well as neurotrophic factors, including brain-derived neurotrophic factor (BDNF) and insulin-like growth factor-1, which promote tissue repair and support neuronal survival. Therefore, modulation of microglial activation represents an additional therapeutic strategy for attenuating neuroinflammation. This perspective extends beyond the traditional neuron-centered approach by recognizing the contribution of microglial responses to limiting tissue damage and promoting neuronal recovery.

Results from experimental studies have demonstrated that, among other effects, 1,8-cineole has the ability to modulate microglial activation [44,46,47,86]. In an experimental rat model of spinal cord injury (SCI) and a conditional co-culture system, 1,8-cineole modulated the PI3K/AKT and p38 MAPK signaling pathways to inhibit microglial activation and pyroptosis, thereby exhibiting neuroprotective effects on motor neurons [86]. Furthermore, Xu et al. [47] detected that an intraperitoneal injection of 1,8-cineole 1 h before and 30 min after subarachnoid hemorrhage in rats can diminish neuroinflammation by suppressing microglial activation and the production of pro-inflammatory cytokines.

Additionally, some studies examining the effects of essential oils containing high percentages of 1,8- cineole, have obtained similar results. In an in vitro study, Moreira et al. [87] demonstrated that essential oil from *Eucalyptus globulus* leaves, containing 72.3% of 1,8-cineole, reduced the mRNA expression of *Nos2*, *Il-1*β, *Il-6*, *Tnf-α*, and *Ptgs2/Cox-2* in a lipopolysaccharide and Aβ-stimulated BV2 microglial cell line. Similarly, essential oils from *Alpinia officinarum* Hance, a plant in the ginger family, inhibited BV2 microglial cell proliferation, despite differences in their 1,8-cineole content. Although the authors attributed this effect primarily to 1,8-cineole, they emphasized the possibility of the involvement of other essential oil constituents [46,88].

However, neuroinflammation induced by microglia should be viewed in a broader context. While microglia serve as primary sensors of CNS homeostasis, their activation also influences neighboring astrocytes. Astrocytes are the most abundant glial cells and they are essential in maintaining CNS homeostasis. They support the BBB, regulate neurotransmitter uptake and provide metabolic and structural support for neurons. It has been shown that activated microglia can induce reactive astrogliosis via secretion of cytokines, such as interleukin-1 alpha and TNFα, or complement protein 1q [89].

Additionally, astrocytes actively participate in synaptic transmission, neuroinflammation, and the brain’s response to injury and disease [90]. In the state of neuroinflammation, astrocytes change their morphology and function, becoming reactive, which is presented with upregulation of glial fibrillary acidic protein [91]. Like microglia, astrocytes can also be classified as neurotoxic or neuroprotective, and produce proinflammatory or anti-inflammatory mediators, respectively [92]. Astrocyte-derived cytokines and chemokines can contribute to neuronal and oligodendrocyte cell death [75,89]. Reactive astrocytes have been detected in numerous neurodegenerative diseases such as HD, AD and PD as well as ALS, and multiple sclerosis [89].

Studies investigating protective effects of 1,8-cineole on astrocytes are limited, and to our knowledge, only one such study has been published to date. To evaluate this effects, Porres-Martínez et al. [93], pretreated human astrocytoma U373-MG cells with this monoterpene–derived from *Salvia lavandulifolia* Vahl. (Spanish sage)—before inducing oxidative stress with hydrogen peroxide. This pretreatment significantly increased cell viability and prevented morphological changes in astrocytes, specifically, the loss of ramification. Additionally, 1,8-cineole prevented the formation of ROS by 30—45%, depending on the concentration used. It also boosted antioxidant defense via increased expression of transcriptional factor nuclear factor-erythroid 2 p45-related factor 2 (Nrf2) as well as antioxidant and detoxifying enzymes, including catalase (CAT), superoxide dismutase (SOD), glutathione peroxidase (GPx), glutathione reductase, and heme oxygenase-1 (HO-1).

A recent study documented that 1,8-cineole prevents cerebral edema in mice infected with malaria [94]. Even though the authors did not directly link these effects to astrocytes, and the research is not directly connected to the previous studies on 1,8-cineole and neurodegeneration, it could be of great importance to examine the role of astrocytes in this context. It is known that astrocytes contribute edema formation through aquaporin-4 water channels and undergo apoptotic changes in advanced BBB models of *P. falciparum* [95]. Testing 1,8-cineole in such a 3D BBB model could provide valuable insights into potential protective effects on astrocytes.

The interaction between 1,8-cineole and nuclear factor kappa B (NF-κB) is of particular interest across diverse cell types, including glial cells, given the pivotal role of NF-κB as a key regulator of inflammatory signaling in the CNS. NF-κB is a dimeric protein complex that regulates DNA transcription, innate and adaptive immune responses, cell proliferation, and differentiation. In the resting state, NF-κB is located in the cytoplasm, inactivated and bound to the inhibitor of nuclear factor kappa B (IκB) [96]. Upon canonical NF-κB pathway activation by cell-specific immunomodulatory factors, IκB is phosphorylated and targeted for proteasomal degradation. This liberates NF-κB in the cytoplasm and enables its translocation to the nucleus to regulate transcription of hundreds of target proinflammatory genes [97,98,99]. Activation of NF-κB in microglia [100] and astrocytes [101] is associated with exacerbation of neuroinflammation and progression of neurodegeneration [102]. On the other side, its activation in neurons has related to synaptic plasticity and cognitive improvement [103].

Many natural terpenoids have been identified as inhibitors of NF-κB signaling with anti-inflammatory potential [104]. A study by Greiner et al. [43] identified multiple molecular mechanisms by which 1,8-cineole affects the NF-κB signaling pathway in U373 glioblastoma cells. This monoterpene inhibited the translocation of the p65 subunit of NF-κB into the nucleus, thereby preventing transcription of pro-inflammatory genes. Moreover, 1,8-cineole stabilized the interaction between NF-κB and IκBα, preventing their dissociation in the cytoplasm. Subsequent retention of NF-κB in the cytoplasm led to decreased production of inflammatory proteins. Additionally, 1,8-cineole diminishes the activation of c-Jun N-terminal kinase 1/2, an enzyme critical for the production of transcription factor Activator Protein-1 [43], which further modulates NF-κB signaling [105].

The effects of Aβ induced inflammation on PC-12 neuronal cells were investigated in a study by Khan et al. [44]. The authors demonstrated that pretreatment with 1,8-cineole significantly downregulates NF-κB protein expression. This suppression subsequently reduces the levels of downstream enzymes cyclooxygenase-2 and inducible nitric oxide synthase. Furthermore, the secretion of major pro-inflammatory cytokines, specifically TNF-α, IL-1β, and IL-6—diminishes, thereby protecting the neuronal cells from Aβ toxicity [44]. In a study by Zhou et al. [86], 1,8-cineole modulated p38 MAPK and PI3K/AKT signaling pathways, which act as upstream regulators of NF-κB, promoting recovery after SCI. Blocking these pathways prevents NF-κB nuclear translocation, halting NLRP3 inflammasome assembly and pro-inflammatory cytokine production. Consequently, suppressing this cascade blocks microglial pyroptosis and averts neuronal loss [86].

### 3.2. Antioxidant Defense Activation, Enzymatic Regulation, and ROS Scavenging

Disturbance in the equilibrium of ROS production and antioxidant system function results in oxidative stress [106]. In neural tissue, overproduction of ROS and persistent oxidative stress are largely responsible for continuous neuronal loss and alterations in neuron-glia connectivity and represent a foundation for the progressive cognitive function decline seen in neurodegenerative disorders [107]. Studies have shown that oxidative stress is involved in the pathogenesis of ALS, AD, and PD [108] and the progression of ischemic brain damage [41].

The transcription factor Nrf2 is a key redox regulator, ubiquitously present in various organs, including the brain [107]. Nrf2 reduces oxidative stress and protects against cellular damage by promoting the expression of antioxidant enzymes SOD, HO-1, glutathione (GSH), CAT, uridine 5′-diphospho-glucuronosyltransferase (UDP-glucosyltransferase) [109,110]. Under basal conditions, Nrf2 is retained in the cytoplasm through its association with Kelch-like ECH-associated protein 1 (Keap1) [111]. Oxidative stress disrupts this interaction, allowing Nrf2 to translocate to the nucleus and induce the expression of antioxidant genes [112]. Nrf2 stability is also regulated by glycogen synthase kinase-3β (GSK3-β), which promotes its proteasomal degradation [113,114]. The PI3K/AKT survival pathway can inhibit GSK3-β through phosphorylation, thereby preventing Nrf2 degradation and enhancing its nuclear translocation [115]. Activation of the Nrf2/Keap1 antioxidant system supports normal mitochondrial function, helping cells to protect themselves from oxidative damage and meet the brain’s high energy demands and antioxidant requirements [115].

Studies have shown that 1,8-cineole promotes the Nrf2 signaling pathway in various models of neurological damage. By promoting its activation and nuclear translocation, 1,8-cineole may enhance Nrf2 binding to antioxidant response elements, thereby upregulating downstream antioxidant and cytoprotective genes. This mechanism may contribute to the enhanced antioxidant defense observed following 1,8-cineole treatment. This was demonstrated in an in vitro ischemia-hypoxia-reperfusion model of hippocampal HT22 neurons, in which 1,8-cineole promoted the activation and nuclear translocation of Nrf2, ultimately leading to increased antioxidant defense. Consistent with these findings, in an in vitro oxygen-glucose deprivation/reoxygenation (OGD/R) model of ischemia using cortical neuron/glia culture, pretreatment with 1,8-cineole increased SOD activity [41]. Similarly, pretreatment of pheochromocytoma PC-12 cells of neural origin with 1,8-cineole prevented cell loss and morphological changes by enhancing the activity of this antioxidant system. This was evident from the significant nuclear translocation of Nrf2 and the upregulation of the antioxidant enzymes such as: CAT, SOD, GPx, glutathione reductase, and HO-1 [48].

In a rat model of subarachnoid hemorrhage 1,8-cineole activated the Nrf2/HO-1 antioxidant pathway, as indicated by increased Nrf2 and HO-1 protein expression following oxidative stress induced by experimental injury [47]. Similarly, in a mouse model of chronic unpredictable mild stress, 1,8-cineole activated the PI3K/AKT/Nrf2 signaling pathway, promoting Nrf2 nuclear translocation, restoring antioxidant defense enzymes such as HO-1 and SOD, and reducing ROS accumulation. The involvement of Nrf2 in the protective effects of 1,8-cineole was further supported by a loss-of-function experiment in corticosterone-induced nerve-like PC-12 cells. Silencing Nrf2 expression using siRNA abolished the protective effects of 1,8-cineole, indicating that Nrf2 signaling is functionally involved in these antioxidant and cytoprotective effects [116].

In addition to the potential of 1,8-cineole to enhance antioxidant system defense, several in vitro studies have shown that it also acts as a free radical scavenger. For example, Porres-Martínez et al. [48], reported that pretreatment of PC-12 cells with 1,8-cineole reduced ROS production.

### 3.3. Modulation of Central Neurotransmitter Systems and Synaptic Plasticity

Besides neuronal loss, neurotransmission deregulation can influence the development, progression, and symptom onset of different neurodegenerative diseases [117]. Moreover, neurotransmission and neuroinflammation are highly interdependent processes, and disturbances in neurotransmission systems among various brain cells can play an essential role in the development of neuroinflammation [118]. For example, neurotransmitters can modulate microglial activity, while microglial inflammatory mediators can directly alter synaptic signaling and neuronal excitability [119]. Neurotransmission modulating drugs, which aim to restore the chemical balance and alleviate symptoms, have been used for years as the standard treatment for AD [120], PD [121], HD, and ALS [122]. For instance, in the treatment of AD, in addition to the anti-amyloid antibodies, antagonism to N-methyl d-aspartate (NMDA) receptors and enhanced cholinergic signaling remain main therapeutic approaches [123].

As 1,8-cineole commonly occurs alongside other terpenoids in essential oils, its biological effects may reflect synergistic, additive, or antagonistic interactions with co-occurring constituents [51]. Multiple in vitro studies have shown that essential oils containing 1,8-cineole as the main compound exert anticholinesterase (AChE) effects [50,51,53,54]. Perry et al. [50] identified 1,8-cineole as a main monoterpene of *S. lavandulaefolia* Vahl. that exerts AChE effects in vitro. Additionally, essential oil of *S. lavandulaefolia* showed in vivo inhibition of AChE in the striatum and hippocampus after oral administration in rats. In the same study, the authors conducted a 6-week pilot trial involving a cohort of patients with mild to moderate AD, who took capsules containing *S. lavandulaefolia* essential oil and observed cognitive improvement. Perry et al. [50] initially implicated, and Savelev et al. [51] subsequently confirmed that the majority of AChE activity in *S. lavandulaefolia* essential oil is due to 1,8-cineole. However, its final effect results from complex synergistic and antagonistic interactions among all constituents of this specific essential oil [51]. Similar results have been observed in other studies. Orhan et al. [53] detected that rosemary oil extracts, containing 1,8-cineole as the main compound (44.42%), inhibit both neuronal AChE and plasma butyrylcholinesterase activities. The authors emphasize that although 1,8-cineole is the most potent monoterpene AChE inhibitor, a synergistic interaction between 1,8-cineole and α-pinene drives the overall observed effect. In their study, Moss et al. [54] confirmed that exposure to rosemary essential oil aroma in healthy individuals leads to detectable levels of 1,8-cineole in the serum. Moreover, these higher concentrations of absorbed 1,8-cineole improved speed and accuracy in cognitive task performance in tested individuals. These findings were associated with enhanced cholinergic neurotransmission. More broadly, recent evidence supports the nootropic potential of terpene-rich essential oils. Lechkova et al. [124] reported improved cognitive function and increased hippocampal BDNF expression following administration of *Tanacetum vulgare* essential oil containing 5.99% of 1,8-cineole.

PD is primarily characterized by the progressive loss of dopaminergic neurons in the *substantia nigra pars compacta* [125]. The consequent loss of dopamine leads to the development of motor symptoms [126], so pharmacological options aim to restore dopamine levels or modulate cholinergic activity [126]. Several preclinical studies have shown evidence that 1,8-cineole increases dopamine levels. Kako et al. [52] reported on increased dopamine release from rat striatum slices after application of *Eucalyptus globulus* essential oil and 1,8-cineole, as its most abundant component. Additionally, the effect of 1,8-cineole on dopamine levels was confirmed in a mouse pilocarpine seizure model receiving 1,8-cineole before seizure onset. Additionally, an increase in noradrenaline levels was also observed [57].

Drugs that modulate gamma-aminobutyric acid (GABA) transmission are widely used for treatment of numerous CNS disorders, such as seizures, anxiety and sleep disturbances. GABA-modulating drugs used in neurodegenerative diseases reduce neuronal firing by causing membrane hyperpolarization and are used as supportive therapy. In AD and ALS, GABA modulation aims to minimalize excitotoxicity and improve synaptic plasticity [127], while in PD and HD regulation of GABA activity can modulate neuronal circuits to relieve motor dysfunction caused by loss of dopamine signaling or degraded inhibitory neurons, respectively [128,129]. 1,8-cineole showed anxiolytic, antidepressant [56] and anticonvulsant [130] effects in several animal models acting through the GABA system. Moreover, the anxiolytic effects were reversed by the GABA receptor antagonist flumazenil, confirming that 1,8-cineole acts on GABA_A_/benzodiazepine receptors [56]. Anxiolytic effects mediated by action on GABA_A_ receptors were confirmed in a study by Ceremuga et al. [55]. Using ambient mass spectrometry imaging in a rat model, Wu et al. [35] detected accumulation of 1,8-cineole and its metabolites in cortex, hippocampus and pineal gland. In these brain regions, the authors observed significant alterations in neurotransmitter metabolites associated with GABAergic and serotoninergic signaling pathways. These findings suggest that the observed sedative–hypnotic effects may be mediated, at least in part, by CNS inhibition [35].

Degeneration of serotonin-producing neurons and/or their receptors occurs early in the course of AD, PD, FTLD, and ALS, often preceding the onset of clinical symptoms [131]. Balanced serotonin levels can modulate mood, sleep, psychiatric symptoms and cognition, but can also influence neuroinflammation and synaptic plasticity [131]. The aforementioned study by Wu et al. [35] detected a significant increase in tryptophan, serotonin, and melatonin levels after the application of 1,8-cineole to rats, indicating increased serotonin synthesis and metabolisms. A noted increase in serotoninergic signaling and 1,8-cineole accumulation was specifically found in the pineal gland, explaining 1,8-cineole’s role in sedative-hypnotic effects [35]. In a study examining monoamine oxidase A inhibition by constituents of ginger (*Zingiber officinale* Roscoe), the authors found that 1,8-cineole inhibits 44.1% of this enzyme activity, emphasizing its antidepressant potential [132].

### 3.4. Inhibition of Apoptotic Neuronal Death

Studies have shown that 1,8-cineole exerts anti-apoptotic effects on injured or stressed cells [40,42,58,59,133]. In contrast, 1,8-cineole has shown consistent pro-apoptotic effects across different cancer cell types [134,135].

Cisplatin therapy used in various cancer treatments induces both central and peripheral neurotoxicity in 50% of the treated patients [136]. In an experimental study examining neuroprotective effects of 1,8-cineole, after cisplatin-induced neuronal loss in rats, a 7-day treatment increased expression of anti-apoptotic gene B-cell lymphoma 2 (Bcl-2) in the left hemisphere prefrontal cortex. Additionally, it amplified antioxidant defense by reducing malondialdehyde (MDA) levels, increasing vitamin C and GSH content, and enhancing glucose-6-phosphate dehydrogenase and CAT activity [59]. In the pilocarpine model of epilepsy in rats, Rad et al. [58] detected increased Bcl-2 mRNA expression, decreased caspase-3 mRNA expression, increased total antioxidant capacity, and reduced apoptotic/necrotic cell counts in the CA1-3 regions of the hippocampus, after oral 28-day treatment with 1,8-cineole. This reduction in pilocarpine-induced cellular death confirmed 1,8-cineole’s neuroprotective potential in a delayed intervention after injury [58].

The neuroprotective properties of 1,8-cineole were documented in an in vivo ischemia rat model by Meng et al. [42]. A 7-day pretreatment reduced the infarct size, neurological dysfunction and neural death in the ischemic region. The authors report that such effects are a consequence of 1,8-cineole’s ability to inhibit the transient receptor potential canonical 6 channel (TRPC6), αII-spectrin degradation, and activation of CREB via MEK/CREB and CaMKIV/CREB signaling pathways, which increased cell survival [42].

Prevention of neuronal loss has also been demonstrated in both in vitro and in vivo AD models by An et al. [40]. Interestingly, a 28-day oral administration of 1,8-cineole preserved neuronal function by protecting mitochondrial membrane integrity, indicating multi-target neuroprotective mechanisms [40]. Using a specifically formulated cyclodextrin encapsulation of 1,8-cineole, in a transgenic *Caenorhabditis elegans* model of AD, Tan et al. [133] demonstrated that 1,8-cineole induces autophagy, an important cellular waste management system, frequently disrupted in neurodegenerative disorders [137].

### 3.5. Regulation of Ion Channels Involved in Pain Modulation

Patients with neurodegenerative disorders frequently experience various types of pain, although the underlying mechanisms and origins may differ. Studies investigating pain frequency report a high prevalence ranging from 8 to 75% in AD, 40% to 86% in PD, and 19 to 85% in motor neuron diseases, depending on the method used to assess pain and the patient’s willingness to report it [138].

Animal studies evaluating effects of 1,8-cineole in pain management have reported promising results [39,139]. Zhang et al. [139] demonstrated that chronic constriction injury of the sciatic nerves upregulates P2X purinergic receptor 3 (P2X3) in dorsal root ganglia, an ion channel that plays an important role in nociception. However, oral 1,8-cineole downregulated P2X3 receptor transcription and protein levels, directly suppressing peripheral pain signaling and subsequent inflammatory damage, as confirmed by behavioral tests [139]. The same group further contributed to our understanding of pain transmission by replicating injury conditions in rats and investigating the effect of 1,8-cineole on P2X purinergic receptor 2 (P2X2) receptors [39]. Downregulation of P2X2 mRNA and protein levels was detected in the dorsal horn of the spinal cord, confirming the central analgesic effect of 1,8-cineole in pain signaling [39].

In contrast, Jiang et al. [60] reported opposing findings. Authors found that 1,8-cineole acts as an agonist at the transient receptor potential ankyrin 1 (TRPA1) ion channel, reducing high-threshold C-fiber pain transmission and enhancing glutamate transmission in *substantia gelatinosa* neurons of rat spinal cord slices [60]. Additionally, similar findings were reported in a study of adult rat spinal slices, in which 1,8-cineole increased spontaneous excitatory postsynaptic currents in *substantia gelatinosa* neurons in a concentration-dependent manner. This effect was caused by opening presynaptic TRPA1 calcium channels in sensory neurons, thereby stimulating excitatory neurotransmitter release [140].

NMDA receptors in the spinal dorsal horn contribute to central sensitization by enhancing glutamatergic signaling from primary afferent neurons, thereby playing an important role in the development and maintenance of chronic and neuropathic pain [141]. However, in neuron/glia cultures, 1,8-cineole reduced cell death following NMDA induced injury, not by directly blocking the NMDA pathway but by boosting antioxidant defense [41].

1,8-cineole does not act through a single specific receptor. Due to its highly lipophilic nature, it can directly modulate ion channels, including TRPA1 and P2X receptors, thereby influencing neurotransmission at both presynaptic and postsynaptic sites. This may trigger a cascade of downstream effects involving multiple neurotransmitter systems and their interactions. In addition, the receptor-independent antioxidant properties of 1,8-cineole may provide neuroprotective effects by reducing oxidative damage, regardless of the neurotransmitter involved.

## 4. Therapeutic Potential and Evidence for 1,8-Cineole in Central Nervous System Disorders

This section reviews current evidence regarding the effects of 1,8-cineole in major neurodegenerative diseases, including AD and PD, as well as brain injuries like ischemic stroke and TBI, both of which may contribute to long-term neurodegeneration. It also discusses the potential therapeutic benefits of 1,8-cineole for anxiety and depression, which may manifest as early symptoms or common neuropsychiatric complications of neurodegenerative diseases.

### 4.1. Therapeutic Potential and Evidence of 1,8-Cineole in Alzheimer’s Disease

As a neurodegenerative disorder, AD is characterized by the progressive accumulation of extracellular Aβ plaques, intracellular deposition of hyperphosphorylated tau protein, chronic neuroinflammation, oxidative stress, and progressive synaptic dysfunction, all of which contribute to neuronal loss and cognitive decline [142]. These pathological processes are closely interconnected, as oxidative stress promotes Aβ production and tau hyperphosphorylation, whereas Aβ accumulation further enhances neuroinflammatory signaling and neuronal injury, creating a self-perpetuating cycle of neurodegeneration [143]. Although disease-modifying therapies targeting amyloid pathology have recently become available, their clinical benefits remain modest and are accompanied by significant limitations, highlighting the need for complementary therapeutic strategies that target multiple pathological processes [144]. Consequently, increasing attention has been directed toward natural compounds capable of simultaneously modulating several pathogenic mechanisms rather than targeting a single molecular pathway [69].

Although available evidence remains limited, experimental studies consistently suggest that 1,8-cineole exerts neuroprotective effects across several key pathological features of AD (Table 1).

For example, in neuronal cells exposed to Aβ, pretreatment with 1,8-cineole improved cell survival while attenuating oxidative stress and inflammatory responses. These protective effects were accompanied by reduced production of reactive oxygen and nitrogen species, suppression of pro-inflammatory cytokines, preservation of mitochondrial function, and restoration of cholinergic activity, indicating that simultaneous modulation of neuroinflammation and oxidative damage may represent one of the principal mechanisms underlying its neuroprotective action [44].

Beyond limiting secondary neuronal damage, 1,8-cineole may also interfere with the early stages of amyloid pathology. In vitro studies demonstrated that it reduces the formation of Aβ42 oligomers and exhibits potent free radical-scavenging activity. However, 1,8-cineole appears less effective than cardamom extracts containing additional bioactive monoterpenes, suggesting possible synergistic interactions between these phytochemicals. Importantly, 1,8-cineole did not disaggregate pre-existing amyloid oligomers, indicating that its beneficial effects are more likely associated with preventing amyloid formation than reversing established deposits [145]. Given the high stability of Aβ42 oligomers, it is consistent with current literature that 1,8-cineole acts as a preventive rather than a therapeutic agent for established pathology.

Additional evidence suggests that the neuroprotective effects of 1,8-cineole extend beyond its antioxidant properties. In an AD model induced by advanced glycation end products, treatment with 1,8-cineole reduced both Aβ production and tau hyperphosphorylation while attenuating oxidative stress, indicating simultaneous modulation of the two principal neuropathological hallmarks of the disease [40]. Mechanistically, these effects were associated with the inhibition of enzymes involved in amyloid generation and pathological tau phosphorylation, supporting the concept that 1,8-cineole may modulate multiple pathogenic pathways involved in AD progression rather than acting through a single molecular target [40].

Evidence from animal models of AD remains relatively scarce but supports these observations. Besides reducing core AD-like pathological changes in an advanced glycation end product-induced rodent model, recent work in a transgenic *Caenorhabditis elegans* model demonstrated that 1,8-cineole delayed Aβ-induced paralysis, reduced amyloid aggregation, enhanced endogenous antioxidant defenses, and promoted autophagy pathways critical for proteostasis [133]. While behavioral evidence from mammalian AD models is currently limited, these findings provide preliminary support that the molecular effects observed in vitro may translate into functional neuroprotection in vivo.

Overall, the current evidence indicates that 1,8-cineole acts as a promising multi-target therapeutic candidate for AD, capable of simultaneously counteracting key pathological drivers, including oxidative stress, neuroinflammation, amyloid pathology, and tau-related abnormalities. However, the available literature remains limited to a small number of preclinical studies, and additional investigations using well-characterized transgenic AD models with comprehensive cognitive and behavioral assessment are required before its therapeutic potential can be adequately evaluated.

### 4.2. Therapeutic Potential and Evidence of 1,8-Cineole in Parkinson’s Disease: Dopaminergic Neuroprotection and Motor Recovery

PD is characterized by progressive degeneration of dopaminergic neurons in the *substantia nigra pars compacta* and the subsequent loss of striatal dopamine, resulting in the characteristic motor symptoms of bradykinesia, rigidity, tremor, and postural instability. Non-motor symptoms like depression, dementia, sleep disorders, etc., are also common symptoms of PD [146]. The pathophysiological background involves oxidative stress, mitochondrial dysfunction, impaired protein homeostasis, chronic neuroinflammation, and abnormal α-synuclein aggregation, which collectively contribute to neuronal injury and disease progression [146,147,148]. These mechanisms make multi-target compounds that could simultaneously modulate oxidative, inflammatory, mitochondrial, and protein-aggregation pathways, such as 1,8-cineole, interesting as a potential therapeutic approach for PD [146,147] (Table 2).

The direct evidence for the therapeutic potential of 1,8-cineole in PD comes from a bilateral 6-hydroxydopamine rat model [37]. Treatment with *Eucalyptus globulus* essential oil containing 81.8% 1,8-cineole improved motor performance, muscle strength, learning, and spatial memory, while reducing neurodegenerative and inflammatory changes. Importantly, the treatment increased the expression of tyrosine hydroxylase and dopamine transporter in the caudate-putamen, suggesting preservation of nigrostriatal dopaminergic function. These findings provide preliminary evidence that a 1,8-cineole-rich preparation may influence both motor deficits and the underlying dopaminergic pathology of PD. However, because the study used a complex essential oil rather than purified 1,8-cineole, contributions from other constituents cannot be excluded [37].

Oxidative stress and mitochondrial dysfunction are relevant to PD because dopaminergic neurons are highly vulnerable to ROS and metabolic stress. Consistent with that, 1,8-cineole has demonstrated the ability to reduce ROS and lipid peroxidation and restore endogenous antioxidant defenses in both in vitro [41] and animal models [49,116]. These effects involve activation of antioxidant pathways including PI3K/AKT/Nrf2 signaling and preservation of mitochondrial function [49,116]. Although these findings originate predominantly from non-PD models and are discussed in greater detail elsewhere in this review, they provide mechanistic support for the potential protection of dopaminergic neurons against oxidative, mitochondrial, and apoptotic injury.

Neuroinflammation represents another important component of PD progression, particularly through persistent activation of microglia and NF-κB-dependent inflammatory signaling. 1,8-cineole inhibited NF-κB activation and increased PPAR-γ expression in an LPS-induced inflammatory model in mice [151], while direct inhibition of NF-κB p65 nuclear translocation was demonstrated in cellular models of inflammation, including astroglial cells [43]. Furthermore, 1,8-cineole reduced inflammatory cytokine production together with inducible nitric oxide synthetase, cyclooxygenase-2, and NF-κB expression in neuronal cells exposed to Aβ-induced injury [44], as previously mentioned. Additional evidence for coordinated PPAR-γ- and Nrf2-associated anti-inflammatory and antioxidant effects was obtained in experimental colitis [152]. Complementary evidence from *Salvia officinalis* essential oil containing 1,8-cineole demonstrated antioxidant activity together with inhibition of 5-lipoxygenase and xanthine oxidase, further supporting the potential of 1,8-cineole-containing preparations to modulate oxidative and inflammatory processes [153]. Together, these findings suggest that 1,8-cineole may attenuate inflammatory processes that contribute to progressive dopaminergic neuronal loss.

Importantly, 1,8-cineole may also influence α-synuclein pathology, a central molecular feature of PD. In a whole-cell biosensor, purified 1,8-cineole reduced α-synuclein aggregation in a concentration-dependent manner, with approximately 50% and 75% reductions observed at 10 and 100 μM, respectively, providing preliminary evidence of a potentially PD-relevant anti-aggregation effect [149]. Previously, an in vitro study also demonstrated that purified 1,8-cineole did not enhance α-synuclein fibrillation but instead inhibited fibrillation when tested at 2% (*v*/*v*) [150]. In contrast, *Myrtus communis* essential oil, which contains 1,8-cineole among its major constituents, markedly increased α-synuclein fibrillation and the formation of toxic aggregates, suggesting that the effects of complex essential oils cannot necessarily be attributed to their major individual components and may depend on interactions among multiple constituents [150].

Overall, the available evidence suggests that 1,8-cineole may have therapeutic relevance to PD through a combination of antioxidant, mitochondrial, anti-inflammatory, and potentially anti-aggregation mechanisms. However, direct PD-specific evidence remains limited to a small number of studies, including a bilateral 6-hydroxydopamine rat model, in which 1,8-cineole was associated with improvements in functional and dopaminergic outcomes in the context of 6-hydroxydopamine-induced neurodegeneration and oxidative stress [37]. Future studies using purified 1,8-cineole in both toxin-induced and α-synuclein-based PD models should determine whether these mechanisms translate into sustained preservation of nigrostriatal neurons, reduced pathological α-synuclein accumulation, and long-term improvement of motor function.

### 4.3. Therapeutic Potential of 1,8-Cineole in Ischemic Stroke: Mitigation of the Ischemic Cascade and Cerebral Reperfusion Injury

Ischemic stroke and cerebral ischemia-reperfusion injury represent a cascade of interconnected pathological events in which cerebral edema, vascular dysfunction, and oxidative tissue damage during reoxygenation actively reinforce one another [154,155]. A small but consistent body of preclinical research suggests that 1,8-cineole may help target all three components of the injury (Table 3).

Cerebral edema following ischemia-reperfusion injury develops through two temporally distinct but overlapping mechanisms. Cytotoxic edema arises within minutes of vessel occlusion. ATP depletion causes failure of the Na^+^/K^+^-ATPase, leading to intracellular sodium and water accumulation and neuronal swelling, while vasogenic edema evolves over hours due to the degradation of tight junction proteins in BBB by matrix metalloproteinases, ROS, and calcium-activated proteases [154,155]. Central to this process is the pathological activation of calpain, a calcium-dependent cysteine protease. Intracellular Ca^2+^ overload activates calpain, cleaves cytoskeletal proteins such as αII-spectrin and degrades non-selective cation channels, including TRPC6. The resulting disruption of CREB-mediated survival signaling reduces neuroprotection and contributes to cellular swelling. Vasogenic edema evolves over subsequent hours as calpain and other proteases, together with matrix metalloproteinases and ROS, degrade tight junction proteins of the BBB, allowing plasma proteins and fluid to extravasate into the brain parenchyma [154,155].

In a rat middle cerebral artery occlusion model, Meng et al. [42] demonstrated that 7-day oral 1,8-cineole pre-treatment (100 mg/kg) significantly reduced infarct volume and suppressed αII-spectrin breakdown product 145 formation, a validated calpain-specific proteolytic fragment of αII-spectrin. Concurrently, TRPC6 expression was preserved in ischemic tissue, maintaining this channel’s capacity to activate downstream CREB-mediated neuroprotective pathways. Brain water content was not independently measured; however, the combination of reduced infarct volume, suppressed calpain activity, and preserved TRPC6 expression suggests anti-edematous effect, primarily at the cytotoxic level [42]. Neurological deficit scores were correspondingly improved, confirming that the biochemical findings translate into functional benefit in this model.

Nrf2-mediated cytoprotection promotes neuronal survival and, consequently, contributes to the preservation of neurovascular unit integrity. Liu et al. [49] showed that 1,8-cineole, at an optimal concentration, fully restored mitochondrial membrane potential and significantly increased mitochondrial ATPase activity in HT22 cells (murine hippocampal neuronal line) exposed to OGD/R. This indicates preserved mitochondrial bioenergetics under reoxygenation stress. The underlying mechanism was traced to the nuclear translocation of Nrf2 and the transcriptional upregulation of its target genes, namely *HMOX1* and *NQO1*. 1,8-cineole treatment significantly lowered lipid peroxidation and MDA levels compared to OGD/R controls. Notably, all protective effects were abolished by the selective Nrf2 inhibition, confirming that the observed cytoprotection reflects pathway-specific activation rather than non-specific radical quenching. In the same study, Liu et al. [49] also reported reduced DNA, protein, and oxidative lipid damage, together with decreased lactate dehydrogenase leakage, indicating preserved membrane integrity. Complementary, in vivo evidence from Wu et al. [116] confirmed dose-dependent activation of the PI3K/AKT/Nrf2 axis, establishing that Nrf2-linked antioxidant and neuronal protection is a reproducible pharmacological feature of 1,8-cineole across model systems rather than a cell-line artefact.

Reoxygenation following cerebral ischemia paradoxically exacerbates tissue damage through a burst of ROS that overwhelm endogenous antioxidant defenses, initiating lipid peroxidation, protein carbonylation, DNA oxidation, and inflammatory activation [116,158]. ROS also reactivates calpain after ischemic calcium overload, creating a feed-forward cycle of oxidative and proteolytic damage that accelerates neuronal death. Ryu et al. [41] provided in vitro evidence that 1,8-cineole intercepts this burst. 1,8-cineole significantly reduced OGD/R-induced cellular injury and suppressed ROS production via two complementary mechanisms: direct free-radical scavenging and enhancement of SOD activity. Importantly, 1,8-cineole also attenuated NMDA receptor–mediated ROS production, a secondary source of reoxygenation-induced oxidative stress distinct from mitochondrial ROS leakage, without inhibiting NMDA-induced calcium influx. This suggests that 1,8-cineole acts after calcium enters the cell, helping to avoid the excitotoxic effects that can result from directly blocking NMDA receptors [159]. These findings are consistent with the broader antioxidant and cytoprotective effects reported by Liu et al. [49]. Taken together with reduced infarct volume and direct edema reduction, these data support its convergent antioxidative, anti-proteolytic, and mitochondria-preserving effects [42,156]. Despite this consistency, key limitations remain. Direct edema endpoints (brain water content, Evans blue extravasation, and tight junction protein quantification) have not been assessed in any 1,8-cineole treated ischemia-reperfusion model. Furthermore, all available in vivo studies employ a prophylactic pre-treatment approach, whereas clinical relevance requires documented post-reperfusion efficacy [159,160,161]. These gaps represent the most pressing priorities for translational development of 1,8-cineole in stroke models.

### 4.4. Traumatic Brain Injury (TBI): Reduction in Secondary Brain Damage and Neurovascular Breakdown

TBI is one of the leading causes of mortality and disability globally. It is characterized by an initial primary injury, which is the mechanical impact, followed by a series of secondary injuries that may persist for extended periods, ranging from hours to years’ post-impact. These secondary injury mechanisms, which include neuroinflammation, excitotoxicity, oxidative stress, and disruption of BBB integrity, significantly contribute to the long-term effects of TBI. The complexity and interaction of these processes present substantial challenges for treatment. Current therapeutic approaches primarily aim to stabilize the patient and prevent further injury; however, they often achieve only partial efficacy [162].

Regarding the investigation of 1,8-cineole’s effects on TBI, there is an absence of published preclinical (in vivo/in vitro) or clinical studies. Nevertheless, despite the lack of direct TBI data, 1,8-cineole has exhibited notable neuroprotective efficacy in models of CNS trauma and injury that are closely related, such as SCI and subarachnoid hemorrhage. These models share the fundamental pathophysiological secondary injury cascades observed in TBI.

A recent 2025 study by Zhou et al. demonstrated that 1,8-cineole significantly improved motor function recovery in rats with SCI [86]. It reduced microglial pyroptosis, a form of inflammatory cell death mediated by the NLRP3 inflammasome and inhibited neuronal apoptosis. 1,8-cineole achieved this effect via the modulation of PI3K/AKT and P38 MAPK signaling pathways, thus suppressing neuroinflammation and supporting neuronal regeneration.

In a rat model of subarachnoid hemorrhage, 1,8-cineole (given at a dose of 100 mg/kg, i.p.) markedly reduced brain edema, neurological deficits, and neuronal apoptosis. It significantly downregulated pro-inflammatory cytokines (TNF-α, IL-1β, IL-6) and oxidative stress markers (MDA), while upregulating antioxidant defenses proteins (Nrf2, HO-1, SOD, GPx) and anti-apoptotic protein Bcl-2 [47].

As previously discussed, pretreatment with 1,8-cineole significantly reduced brain infarct size, neuronal death, and neurological deficits in a rat model of ischemic stroke by preventing degradation of TRPC6 and activating cAMP CREB, suggesting that 1,8-cineole exerts neuroprotective effects by preserving neuronal survival signaling pathways [41]. 1,8-cineole has also been shown to ameliorate OGD/R-induced injury in rat cortical neurons/glia, which was associated with upregulation of SOD activity as well as direct ROS scavenging, further supporting its potential utility in the ischemic components of secondary TBI. In HT22 cells exposed to the same injury model, it has been shown to improve cell survival rate and alleviate oxidative stress. Specifically, it reduced ROS, MDA, lipid peroxidation, protein carbonyl, and 8-hydroxy-2′-deoxyguanosine levels, while enhancing the activity of antioxidant enzymes (SOD, CAT, GPx) and GSH content [49].

Although direct evidence regarding the efficacy of 1,8-cineole in TBI is lacking, the mechanisms identified in related conditions correspond closely with the secondary injury cascades observed in TBI. Investigating 1,8-cineole in the context of TBI is a promising approach, as it aligns with several therapeutic strategies already validated by other phytochemicals, including antioxidant defense, inflammatory suppression, and modulation of cell death [163,164]. Previous studies have demonstrated the potential of other phytochemical compounds in TBI treatment. The rationale for extrapolating these findings to 1,8-cineole is based on the convergence of pathways rather than the equivalence of compounds. This mechanistic hypothesis enables the examination of the effects of compounds such as curcumin [165], resveratrol [166], N-acetylcysteine [167], and astaxanthin [168], suggesting their comparability to 1,8-cineole due to their consistent action on the same injury biology that 1,8-cineole appears to modulate in other neuroinflammatory models.

### 4.5. Anxiety and Depression: Neuromodulation of Mood Pathways and Stress Resilience

Anxiety is one of the most prevalent psychiatric disorders in adults, affecting approximately 4% of the global population, and with a significant increase in new cases over the last 30 years [169]. Depression is a serious mental condition which affects millions of people worldwide, with symptoms like a depressed mood, loss of interest, cognitive impairment, and sleep disturbances. Anxiety and depression are highly prevalent psychiatric comorbidities in neurodegenerative diseases such as AD and PD, developing as later-stage symptoms, but also as a potential risk factor that may accelerate disease onset and progression [170,171]. Medications given for treatment of these conditions are not effective in all patients and can cause significant side effects [172].

Consequently, there has been an increase in research on preparations containing natural compounds, like 1,8-cineole, as potential therapeutic agents for these disorders. Preclinical studies have demonstrated that 1,8-cineole, as well as essential oils containing 1,8-cineole, caused significant reductions in anxiety and depressive-like behaviors in both healthy mice and various anxiety mouse models [56,173,174,175]. Clinical evidence is currently limited; however, one randomized controlled trial investigated the effects of inhaled 1,8-cineole on preoperative anxiety in patients before undergoing the selective nerve root block. This double-blind study enrolled 62 patients, which were randomized into control group, treated with inhalations of almond oil, or experimental groups, treated with limonene, 1,8-cineole or eucalyptus oil, 1% *v*/*v* in almond oil. 1 mL of each preparation was put on the aroma pad and kept 30 cm away from the participants nose, who inhaled naturally for 5 min. Anxiety was assessed immediately after inhalation using anxiety visual analog scale, the State-Trait Anxiety Inventory, and the Profile of Mood States, before and after the inhalations. The results show that 1,8-cineole caused significant reduction in anxiety across all three measures [176] (Table 4).

The pathophysiology of anxious-like behavior has been associated with increased oxidative stress and neuroinflammation, especially in the hippocampus. Several mechanisms have been proposed to explain 1,8-cineole’s anxiolytic properties. One of the leading hypotheses proposes that it exerts sedative effects through interaction with the benzodiazepine binding site of GABA_A_ receptors, thereby enhancing chloride influx and neuronal hyperpolarization [56,177]. However, due to the evidence that 1,8-cineole also possesses significant antioxidative and anti-inflammatory effects, these properties may equally underlie its anxiolytic action by mitigating anxiety induced by oxidative stress and neuroinflammation [173,174]. More specifically, in a lead-induced anxiety model, hippocampal levels of MDA were elevated, while GSH levels and activity of SOD and GPx were reduced [173]. Treatment with 1,8-cineole restored these oxidative stress markers to levels comparable with those of the control group. It also prevented the lead-induced upregulation of NF-kB expression as well as reduction in expression of sirtuin 1, a nicotinamide adenosine dinucleotide dependent deacetylase, which has also been implicated in anxiety-like behavior. Additionally, 1,8-cineole increased BDNF expression, whose levels are inversely associated with anxiety-like behaviors [173].

Regarding the potential antidepressant effects of 1,8-cineole, research on rat brain striatal slices has shown that it can stimulate dopamine release [52]. Dopaminergic pathways are often overlooked in the treatment of depression, especially with symptoms of anhedonia, and increased dopaminergic activity could therefore improve treatment outcomes [178]. Studies using a chronic unpredictable mild stress model of depression in mice demonstrated that another possible molecular mechanism for 1,8-cineole’s antidepressant effects could be reduction in free radical production and oxidative stress in the hippocampus, thereby preventing neuronal damage. This antioxidative effect appears to be mediated through activation of the PI3K/AKT/Nrf2 signaling pathway [116]. Finally, 1,8-cineole’s capacity to increase BDNF expression, as already reported in the anxiety model [173], may also represent an important mechanism underlying its antidepressant activity, since down-regulation of BDNF is considered a key contributor to the pathophysiology of depression [179].

## 5. Translational Challenges, Formulation Strategies, and Future Perspectives of 1,8-Cineole

1,8-cineole has traditionally been used as a component of herbal preparations; however, due to its diverse biological effects, it has received increasing attention in pharmacological research. Although it is commonly used as an excipient in the pharmaceutical and cosmetic industries [21], its therapeutic potential has thus far been utilized primarily for respiratory disorders [21]. In recent years, increasing attention has been directed toward its potential applications in neuropharmacology. As discussed in Section 2, the BBB permeability of 1,8-cineole makes it particularly well suited to targeting the CNS. Given the increasing burden of neurodegenerative disorders [2], naturally derived compounds such as 1,8-cineole have attracted interest for their potential to modulate multiple pathological processes involved in neuronal injury and degeneration. The therapeutic potential of 1,8-cineole appears to be associated with its effects on several processes implicated in neurodegeneration, including neuroinflammation and glial regulation [43,44,45,47], antioxidant defense activation, neurotransmission and synaptic plasticity alteration [29,30,31,32,33,34,35,36], inhibition of apoptotic death [58,59] and regulation of pain signaling [39,139].

Although the mechanistic convergence of 1,8-cineole’s actions, involving Nrf2, NLRP3, and TRPC6/CREB pathways, appears promising and targets multiple mechanisms, the translational challenges remain consistent with those encountered across the phytochemical-neuroprotection field. These challenges include dosing, BBB kinetics in humans, and the development of standardized clinical formulations. Presently, all available data are derived from preclinical research, specifically cell culture and rodent models. There are no human trials specific to 1,8-cineole for conditions such as AD, PD, neuropsychiatric disorders, TBI, SCI, or stroke. Existing human data pertains predominantly to respiratory indications, with limited data from clinical studies examining anxiolytic and cognitive effects [50,176].

The future role of 1,8-cineole appears highly promising in several specific domains. It may serve as an adjunct antioxidant in acute injuries such as TBI, SCI, and the early post-stroke phase, due to its function as an activator of the Nrf2/AKT pathway. This is particularly relevant as oxidative burst constitutes the primary early pathology in these conditions. In the context of chronic neurodegeneration, particularly AD, 1,8-cineole could potentially be employed as an anti-neuroinflammatory adjunct, owing to its cytokine-lowering effects in amyloid models. As part of a combination therapy, 1,8-cineole could help reduce the accumulation of Aβ [145] and α-synuclein [149,150] deposits. Furthermore, in CNS trauma, such as TBI or SCI, it may be utilized to reduce seizure risk in affected populations.

A significant translational advantage of 1,8-cineole, compared to other naturally occurring compounds, is its prior clinical use. This facilitates repurposing trials, which can progress more rapidly than the development of new phytochemicals, as existing human pharmacokinetic and tolerability data significantly reduce regulatory barriers.

Despite promising preclinical findings, several pharmacological challenges need to be addressed before the neuroprotective potential of 1,8-cineole can be translated into clinical applications. Its physicochemical characteristics—particularly its volatility, lipophilicity, low aqueous solubility, and susceptibility to extensive first-pass hepatic metabolism—can limit its bioavailability and contribute to relatively rapid systemic clearance [66,67,68]. Consequently, maintaining therapeutic concentration over an extended period may be difficult with standard formulations, often necessitating frequent administration or high doses that could increase side-effect risks. To circumvent these pharmacokinetic constraints, recent translational efforts have focused on advanced drug delivery systems and targeted encapsulation strategies, designed to protect the volatile monoterpene, bypass hepatic extraction, and sustain therapeutic exposure within the CNS.

In this context, nanotechnology has emerged as a particularly promising avenue. This rapidly expanding field offers diverse nanoparticle (NP) systems engineered to overcome traditional biopharmaceutical barriers. NP carriers can prolong the residence time of encapsulated compound within CNS tissue through sustained or controlled release and protect the compound from rapid clearance and metabolism [180]. So far, no NP preparation loaded with 1,8-cineole has been investigated for CNS application, but few 1,8-cineole-solid lipid nanoparticle preparations have been studied for other indications, including protection against vascular endothelial injury in rats and wound healing, with positive outcomes in both cases [181,182].

Polymeric nanoparticles are another promising class of nanocarriers, capable of facilitating BBB passage for peptides and macromolecules encapsulated within their matrix. One study used gelatin-based polymeric NPs loaded with cardamom extract, in which 1,8-cineole is the main component, for the treatment of glioblastoma. Results were promising, since the gelatinous coating of the NP enabled prolonged release of the encapsulated extract into the surrounding tissue, resulting in an increased concentration of 1,8-cineole present at the glioblastoma site [183,184]. This finding shows that encapsulation of 1,8-cineole into an NP-based drug delivery system could offer solutions to its metabolism and elimination problems and extend its presence within CNS tissue.

Regarding delivery route considerations, 1,8-cineole, a small, lipophilic, volatile monoterpene, is a natural candidate for intranasal administration due to its potential for direct CNS access. This approach is currently a significant focus of research. The potential of 1,8-cineole for intranasal delivery is being actively investigated within formulation science, with research focusing on the development of microemulsions of eucalyptus oil for this purpose [185]. Furthermore, building on its capacity to interact with the BBB, the transient modulation of barrier integrity induced by 1,8-cineole is particularly noteworthy from a drug-delivery perspective [38]. This localized effect could potentially enhance CNS penetration by therapeutic compounds that otherwise have limited brain access [38,73].

Although in vitro and live animal studies provide encouraging evidence, clinical data remains scarce. Consequently, rigorously designed clinical studies are necessary to elucidate the pharmacokinetic and pharmacodynamic properties of 1,8-cineole, determine suitable dosing regimens, and assess its long-term safety and potential neuroprotective efficacy in humans. Furthermore, the optimal dose remains to be established. Excessive systemic exposure may result in adverse effects, including CNS depression, respiratory depression, and gastrointestinal irritation [22]. In particular, the safety profile of prolonged or high-dose exposure requires further investigation, especially concerning potential CNS and systemic adverse effects.

## 6. Conclusions

Alongside a critical need to identify novel therapeutic strategies, there is a growing imperative to treat neurodegenerative diseases as complex, interconnected dysfunctions within the CNS. Moving beyond traditional neurocentric paradigms requires multi-target approaches that address pathobiological processes across multiple cellular and biochemical levels. In this context, phytomedicine offers a rich source of multi-targeted bioactive candidates, among which the monoterpene 1,8-cineole shows promising potential.

Non-neuronal glial cells, particularly microglia and astrocytes, play important roles in regulating synaptic transmission, maintaining axonal health, and modulating neuroinflammation. Consequently, modulating microglial activation to restore CNS homeostasis represents a promising neuroprotective strategy [186]. Phytochemicals like 1,8-cineole may be particularly relevant in this context due to their ability to simultaneously attenuate pro-inflammatory signaling via NF-κB/NLRP3 inhibition and upregulate endogenous antioxidant defenses via the Nrf2/Keap1 pathway. Together with its established clinical use and ability to cross the BBB, these properties provide a rationale for further investigation of 1,8-cineole in neurodegenerative diseases and CNS trauma.

However, translating these findings into clinical applications will require further well-controlled human studies addressing brain exposure, pharmacokinetic variability, BBB interactions, optimal dosing regimens, and long-term safety. Thus, while 1,8-cineole represents a promising multi-target candidate for neuroprotective strategies, its therapeutic efficacy in neurodegenerative and other CNS disorders remains to be established clinically.

## Figures and Tables

**Figure 1:**
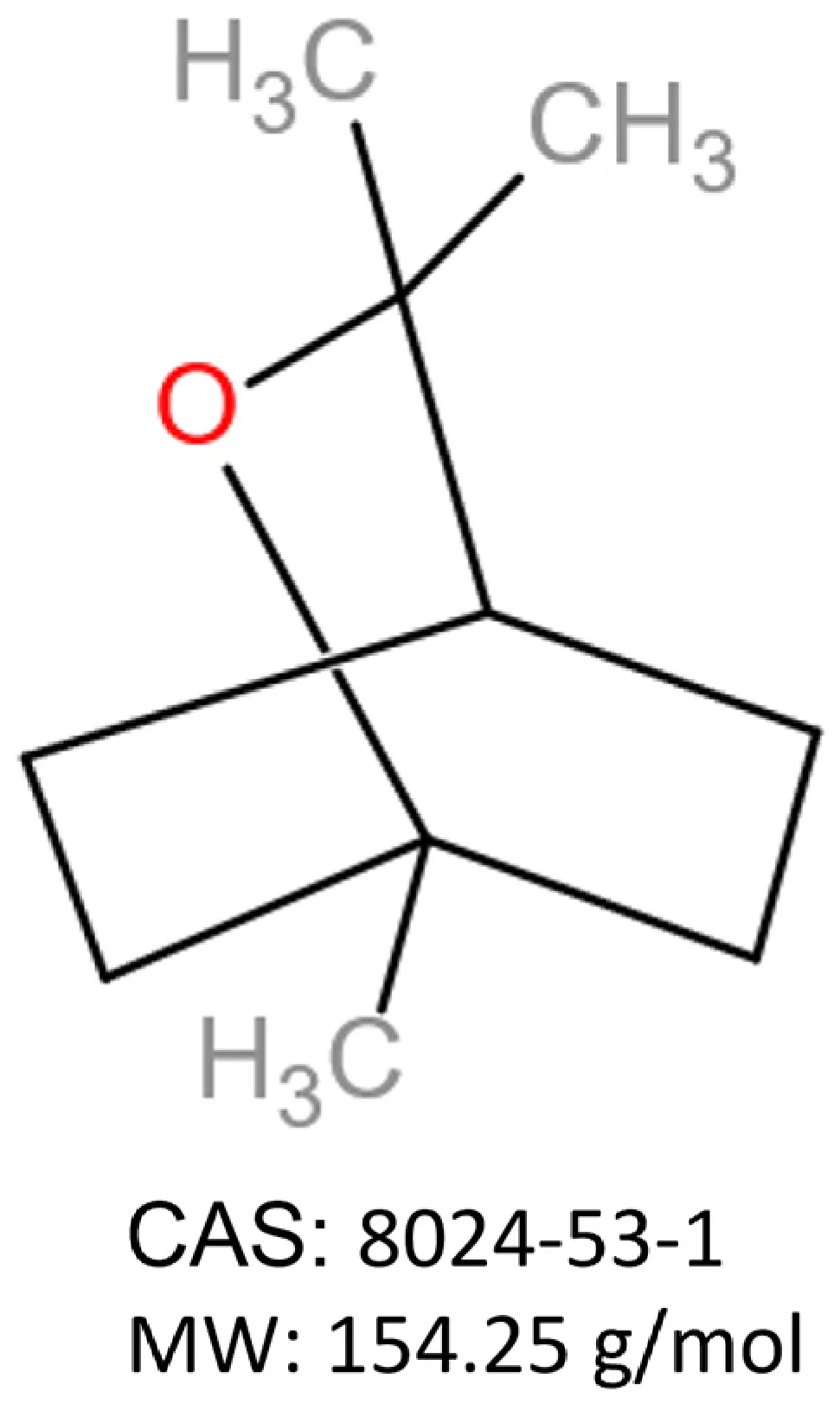
Chemical structure of 1,8-cineole, including its CAS Registry Number and molecular weight (MW).

**Figure 2:**
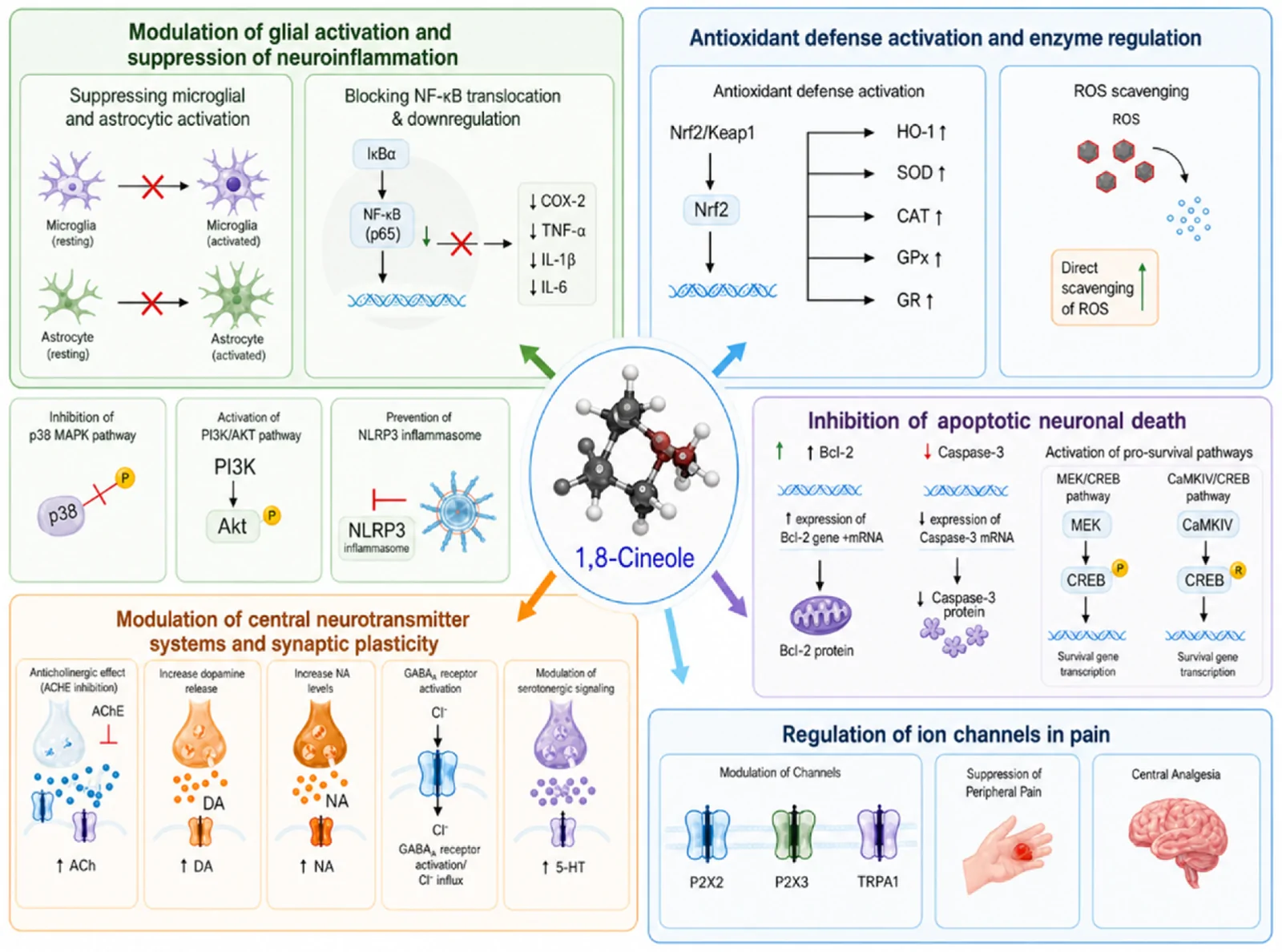
Cellular and Molecular Mechanisms of 1,8-Cineole Action in the Central Nervous System. The figure provides a comprehensive overview of the cellular and molecular mechanisms underlying the effects of 1,8-cineole in the central nervous system (CNS). It illustrates the modulation of several interconnected pathways involved in neuroinflammation, oxidative stress, neuronal survival, neurotransmission and synaptic plasticity, and pain regulation. Abbreviations: NF-κB, Nuclear Factor Kappa B; IκBα, Inhibitor of Nuclear Factor Kappa B Alpha; COX-2, Cyclooxygenase-2; TNF-α, Tumor Necrosis Factor Alpha; IL-1β, Interleukin-1 Beta; IL-6, Interleukin-6; MAPK, Mitogen-Activated Protein Kinase; p38, p38 Mitogen-Activated Protein Kinase; PI3K, Phosphoinositide 3-Kinase; AKT, Protein Kinase B; NLRP3, NLR family pyrin domain containing 3; Nrf2, Nuclear Factor Erythroid 2–Related Factor 2; HO-1, Heme Oxygenase-1; SOD, Superoxide Dismutase; CAT, Catalase; GPx, Glutathione Peroxidase; GR, Glutathione Reductase; ROS, Reactive Oxygen Species; Bcl-2, B-cell Lymphoma 2; Caspase-3, Cysteine-Aspartic Acid Protease 3; MEK, Mitogen-Activated Protein Kinase; CREB, cAMP Response Element-Binding Protein; CaMKIV, Calcium/Calmodulin-Dependent Protein Kinase IV; AChE, Acetylcholinesterase; Ach, Acetylcholine; DA, Dopamine; NA, Noradrenaline; GABA, Gamma-Aminobutyric Acid; GABAA, Gamma-Aminobutyric Acid Type A Receptor; 5-HT, 5-Hydroxytryptamine (Serotonin); P2X2, P2X Purinoceptor 2; P2X3, P2X Purinoceptor 3; TRPA1, Transient Receptor Potential Ankyrin 1; Cl^−^, Chloride Ion; P, Phosphorylated/Phosphate Group.

**Table 1:** Studies Investigating the Effects of 1,8-cineole in Alzheimer’s Disease.

Year	Author	Model	Dose of 1,8-cineol	Main Conclusions
**In vitro**				
2022	An F. et al. [40]	SH-SY5Y human neuroblastoma cells exposed to 500 μg/mL AGEs	5, 10 and 20 μmol/L	Increased cell viability and attenuated AGE-induced cytotoxicity. Prevented AGEs-mediated ΔΨm loss and mitochondrial oxidative damage. Decreased Aβ production through inhibition of BACE-1 activity. Reduced tau hyperphosphorylation by inhibiting AGEs-induced GSK-3β activity, with no effect on PP2A activity.
2019	Paul K. et al. [145]	SHSY5Y cells, oxidative and amyloidogenic stress (Fe^2+^/Ascorbate and Aβ42)	50 and 100 µM	Reduced hydroxyl radical formation, confirming potent antioxidant activity in the Fenton reaction system. Inhibited Aβ42 oligomerization in a concentration-dependent manner, though with slightly lower efficacy compared to the SC-CO_2_ cardamom extract. Demonstrated no significant de-aggregation effect on pre-formed Aβ42 oligomers.
2014	Khan, A. et al. [44]	NGF-differentiated PC-12 cells (rat adrenal pheochromocytoma cell line) toxicated by Aβ25–35 (10 μM)	2.5, 5 and 10 μM pretreatments	CIN pretreatment attenuated Aβ25–35-induced cytotoxicity and effectively restored cell viability. Reduced Aβ25–35-induced oxidative stress through the downregulation of ROS and NO production. Suppressed neuroinflammatory responses by attenuating pro-inflammatory cytokines (IL-1β, IL-6, TNF-α) and inhibiting NF-κB signaling. Protected mitochondrial integrity by restoring MMP and reducing Aβ25–35-induced LDH release. Ameliorated cholinergic dysfunction by restoring ChAT expression in Aβ25–35-treated cells.
**In vivo**				
2024	Tan X. et al. [133]	Transgenic *Caenorabditis elegans*, Genetic expression of human Aβ 1-42 (temperature-dependent)	Dietary supplementation (NGM agar) at 200–1000 µM	Attenuated Aβ-induced paralysis and improved survival under oxidative stress. Reduced Aβ plaque deposition/aggregation and decreased ROS levels. Activated the SKN-1/Nrf2 antioxidant pathway, increasing the expression of downstream antioxidant genes (e.g., GST-4, GCS-1). Induced autophagy by upregulating LGG-1, PHA-4, and UNC-51, contributing to neuroprotection.
2022	An F. et al. [40]	Male Sprague-Dawley rats–AGE-induced AD model	50, 100, or 150 mg/kg (i.g.)	Modulated the AGE/RAGE signaling pathway, thereby mitigating key AD-related pathological changes Prevented the accumulation of 8-OHdG, indicative of reduced oxidative stress Suppressed Aβ accumulation/production and BACE-1 expression Attenuated tau hyperphosphorylation via inhibition of GSK-3β activity.

**Abbreviations:** AD, Alzheimer’s disease; Aβ, amyloid beta; Aβ25–35, amyloid beta fragment 25–35; Aβ42, amyloid beta fragment 42; AGEs, advanced glycation end products; BACE-1, β-site amyloid precursor protein-cleaving enzyme 1 (β-secretase 1); β-CD, beta-cyclodextrin; C/CD-IC, 1,8-cineole/β-cyclodextrin inclusion complex; ChAT, choline acetyltransferase; CIN, 1,8-cineole (eucalyptol); ΔΨm, mitochondrial membrane potential; GCS-1, γ-glutamylcysteine synthetase 1; GSK-3β, glycogen synthase kinase-3 beta; GST-4, glutathione S-transferase 4; i.g., intragastric; IL-1β, interleukin-1 beta; IL-6, interleukin-6; LDH, lactate dehydrogenase LGG-1, GABA receptor-associated protein homolog; MMP, mitochondrial membrane potential; NF-κB, nuclear factor kappa B; NGF, nerve growth factor; NGM, nematode growth medium; NO, nitric oxide; Nrf2, nuclear factor erythroid 2-related factor 2; PC-12, rat adrenal pheochromocytoma cell line; PHA-4, forkhead box A transcription factor homolog; PP2A, protein phosphatase 2A; RAGE, receptor for advanced glycation end products; ROS, reactive oxygen species; SKN-1, skinhead-1 (*Caenorhabditis elegans* homolog of nuclear factor erythroid 2-related factor 2, Nrf2);TNF-α, tumor necrosis factor alpha; UNC-51, unc-51 autophagy activating kinase homolog.

**Table 2:** Studies Investigating the Effects of 1,8-cineole in Parkinson’s Disease.

Year	Author	Model	Dose of 1,8-cineol	Main Conclusions
**In vitro**				
2024	Stylianopoulou et al. [149]	HeLa human cervical carcinoma cells transiently co-transfected with two plasmids encoding α-synuclein fused to complementary fragments of GLuc, generating a whole-cell biosensor for α-synuclein aggregation	1,8-cineole: 10 and 100 µM	1,8-cineole reduced α-synuclein aggregation in a concentration-dependent manner. At 10 µM, aggregation-associated GLuc activity was reduced by approximately 50%, while at 100 µM it was reduced by approximately 75%.
2016	Morshedi and Nasouti [150]	Recombinant human α-synuclein (70 μM) incubated with several essential oils and their major individual constituents under fibrillation-inducing conditions	1,8-cineole: 2% (*v*/*v*); *Myrtus communis* essential oil (0.1, 0.5, 1, 5, and 10% *v*/*v*)	Purified 1,8-cineole inhibited α-synuclein fibrillation. In contrast, *Myrtus communis* essential oil despite containing 1,8-cineole as a major constituent, enhanced α-synuclein fibrillation and promoted the formation of more toxic aggregates.
**In vivo**				
2025	Naranjo-Viteri et al. [37]	Adult male Wistar rats, 8 weeks old; bilateral 6-hydroxidopamine- induced Parkinsonian model	*Eucalyptus globulus* essential oil (81.8% 1,8-cineole): 25 mg/kg i.p.	Improved motor performance, muscle strength, and cognitive deficits like learning and spatial memory. Reduced inflammatory markers (e.g., TNF-α) and neurodegenerative changes suggesting a neuroprotective effect.

**Abbreviations:** GLuc, *Gaussia princeps* luciferase; i.p., intraperitoneal; TNF-α, tumor necrosis factor alpha.

**Table 3:** Studies Investigating the Effects of 1,8-cineole in Stroke and Ischemia–Reperfusion Models.

Year	Author	Model	Dose of 1,8-cineol	Main Conclusion
**In vitro**				
2026	Zhiping et al. [156]	C17.2 NSC line, H_2_O_2_ oxidative stress	6.25, 12.5, 25 μmol/L × 24 h, 12.5 μmol/L (greatest NSC protective effect)	Restored cell morphology and viability of C17.2 NSCs after H_2_O_2_-induced oxidative damage. Preserved cell morphology and significantly increased cell density. Increase in Nestin-positive (neural progenitor) cells. Upregulation of Wnt3a, β-catenin, and Cyclin D1 protein levels.
2023	Liu et al. [49]	HT22 cell line (murine hippocampal neurons), OGDR model	2.5–30 μmol/L screened. 10 μmol/L showed best effect	Reduced OGDR-induced oxidative injury in cortical cells via direct scavenging. Reduced OGDR-induced oxidative damage. Restoring mitochondrial function in HT22 cells via Nrf2/HO-1/NQO1 activation.
2014	Ryu et al. [41]	Primary cortical neuron-glia co-cultures, rat, OGDR	Multiple concentrations screened (0.01–10 μM), 10 μM showed significance	Reduced OGDR-induced oxidative injury in cortical cells via direct scavenging. SOD enhancement, without blocking NMDA receptor calcium influx.
**In vivo**				
2026	Zhiping et al. [156]	Adult male ICR mice, 6–8 weeks, photothrombotic stroke	50, 100, 200 mg/kg *p.o.* × 8 days	Significantly improved spontaneous exploratory behavior in the open-field test. Restored motor coordination and balance on the rotarod test. Improvement of hippocampal histoarchitecture in treated mice. Number of injured neurons was significantly reduced across all hippocampal subregions. Promoted migration of NSCs toward the ischemic region and their differentiation into glial cells and neurons to replace damaged cells.
2021	Meng et al. [42]	Male Sprague-Dawley rats (8–9 weeks old weighing 200–250 g), MCAO models	100 mg/kg for 7 consecutive days	1,8-cineole pretreatment attenuates ischemic stroke-induced brain damage. Blockage of calpain-inducedTRPC6 degradation and activating CREB via MEK/CREB and CaMKIV/CREB signaling pathways.
2020	Farivar et al. [157]	Adult male rats, chronic cerebral hypoperfusion/reperfusion	5 mg/kg, 10 mg/kg, and 20 mg/kg, 10 mg/kg showed significance	Functional cognitive recovery. Neuroprotection under ischemic conditions.

**Abbrevations:** OGD/R, oxygen-glucose deprivation/reoxygenation; ROS, reactive oxygen species; SOD, superoxide dismutase; NMDA, n-methyl-d-aspartat; MDA, malondialdehyde; NSC, Neuronal Stem Cells; Nrf2, Nuclear factor-erythroid 2 p45-related factor 2; HO-1, Heme oxygenase-1; NQO1, NAD(P)H:quinone oxidoreductase 1; MCAO, middle cerebral artery occlusion; TRPC 6, Transient Receptor Potential Canonical 6; CREB, cAMP Response Element-Binding Protein; MEK, Mitogen-Activated Protein Kinase; CaMKIV, Calcium/Calmodulin-Dependent Protein Kinase IV; SAH, Subarachnoid haemorrhage.

**Table 4:** Studies Investigating the Effects of 1,8-cineole on Anxiety and Depression.

Year	Author	Model	Dose of 1,8-cineol	Main Conclusions
**In vitro**				
2008	Kako et al. [52]	Rat brain striatal slices	0.5% 1,8-cineole or *Eucalyptus globulus* essential oil including Krebs buffer, superfusion method	Increased release of dopamine.
**In vivo**				
2025	Asle-Rousta M et al. [173]	Lead-induced anxiety mouse model	100 mg/kg, gavage	Prevented an increase in MDA levels, pro-inflammatory cytokines (TNFα, IL-1β and IL-6) and NF-κB expression. Prevented decrease in GSH levels, SOD and GPxactivity, IL-10 and SIRT1 expression. Increased expression of BDNF improved the performance on EPM and OF.
2025	Wu et al. [116]	CUMS mouse model of depression	50 mg/kg/d, 100 mg/kg/d, and 150 mg/kg/d; orally	Reduction in depression-like behaviors. Better results on SPT, OFT and FST. Reduction in MDA and ROS levels, restoration of antioxidant activity. Improved neuronal structure.
2022	Bahrami et al. [174]	Hyperammonemia rat model	5 and 10 mg/kg of 1,8-cineole, or 15 mg/kg ibuprofen + 10 mg/kg 1,8-cineole; intraperitoneally	Improved EPM results. Increase in SOD1 expression in ibuprofen + 1,8-cineol group.
2020	Dougnon G, Ito M [56]	Mouse model	inhalation administration of doses 4 × 10^−6^ to 4 × 10^−1^ mg per 400 μL of triethyl citrate	Improved LDB and MBT results. Reduced immobility times in the FST and TST. Prevented anxiety and depressive-like symptoms.
2013	Majnooni et al. [175]	Rat model	1 mg/kg of volatile oil with 20.8% 1,8-cineol	Improved EPM results.
2011	Quilez et al. [177]	Mouse model	6 mg/kg and 3.25 mg/kg eucalyptus oil, orally	Inhibition of diazepam’s depressant action.
**Human studies**				
2014	Kim et al. [176]	Randomized Clinical Trial	1,8-cineole or eucalyptus oil,1% *v*/*v* in almond oil; inhalation	Reduced preoperative anxiety Lower A-VAS, STAI and POMS outcomes.

**Abbrevations:** A-VAS, anxiety-visual analog scale; BDNF, brain derived neurotrophic factor; CUMS, chronic unpredictable mild stress; EPM, elevated plus maze; FST, forced-swim test; GSH, glutathione; GPx, glutathione peorxydase; IL-1β, interleukin-1beta; IL-6, interleukin-6; IL-10, interleukin-10; LDB, light-dark box test; MBT, marble-burying test; MDA, malondialdehyde; NF-κB, nuclear factor kappa B; OF, open field test; POMS, profile of mood states; SIRT1, sirtuin 1; SOD, superoxide dismutase; SPT, sucrose preference test; STAI, state-trait anxiety inventory; TNFα, tumor necrosis factor alpha; TST, tail suspension test.

## Data Availability

No new data were created or analyzed in this study. Data sharing is not applicable to this article.

## Abbreviations

The following abbreviations are used in this manuscript:

**Abbreviation**	**Full name**
AD	Alzheimer’s disease
PD	Parkinson’s disease
ALS	Amyotrophic lateral sclerosis
HD	Huntington’s disease
FTLD	Frontotemporal lobar dementia
BBB	Blood–brain barrier
CNS	Central nervous system
ROS	Reactive oxygen species
TBI	Traumatic brain injury
IL-1β	Interleukin-1β
TNF-α	Tumor necrosis factor-α
IL-10	Interleukin-10
IL-6	Interleukin-6
BDNF	Brain-derived neurotrophic factor
SCI	Spinal cord injury
PI3K	Phosphoinositide 3-kinase
AKT	Protein Kinase B
MAPK	Mitogen-activated protein kinase
Aβ	Amyloid-beta
Nrf2	Nuclear factor-erythroid 2 p45-related factor 2
CAT	Catalase
SOD	Superoxide dismutase
GPx	Glutathione peroxidase
HO-1	Heme oxygenase-1
NF-κB	Nuclear factor kappa B
IκB	Inhibitor of nuclear factor kappa
NLRP3	NLR family pyrin domain containing 3
Keap1	Kelch-like ECH-associated protein 1
GSK3-β	Glycogen synthase kinase-3 beta
GSH	Glutathione
OGD/R	Oxygen-glucose deprivation/reoxygenation
NMDA	N-methyl d-aspartate
AChE	Acetylcholinesterase
GABA	Gamma-aminobutyric acid
Bcl-2	B-cell lymphoma 2
MDA	Malondialdehyde
TRPC6	Transient receptor potential canonical 6 channel
CREB	Cyclic adenosine monophosphate response element-binding protein (CREB)
MEK	Mitogen-activated protein kinase
CaMKIV	Calcium/Calmodulin-dependent Protein Kinase IV
P2X3	P2X purinergic receptor 3
P2X2	P2X purinergic receptor 2
TRPA1	Transient receptor potential ankyrin 1
NP	Nanoparticle
